# A new CCCH-type zinc finger-related lncRNA signature predicts the prognosis of clear cell renal cell carcinoma patients

**DOI:** 10.3389/fgene.2022.1034567

**Published:** 2022-09-30

**Authors:** Cheng Shen, Zhan Chen, Jie Jiang, Yong Zhang, Wei Xu, Rui Peng, Wenjing Zuo, Qian Jiang, Yihui Fan, Xingxing Fang, Bing Zheng

**Affiliations:** ^1^ Department of Urology, The Second Affiliated Hospital of Nantong University, Nantong, China; ^2^ Medical Research Center, The Second Affiliated Hospital of Nantong University, Nantong, China; ^3^ Department of Orthopedics, The Second Affiliated Hospital of Nantong University, Nantong, China; ^4^ Department of Paediatric, Chinese Medicine Hospital of Rudong, Nantong, China; ^5^ Department of Pathogenic Biology, School of Medicine, Nantong University, Nantong, China; ^6^ Nephrology Department, The Second Affiliated Hospital of Nantong University, Nantong, China

**Keywords:** CCCH-type zinc finger, lncRNAs, clear cell renal cell carcinoma, immune infiltration, drug therapy

## Abstract

**Background:** Clear cell renal cell carcinoma (ccRCC) is the main component of renal cell carcinoma (RCC), and advanced ccRCC frequently indicates a poor prognosis. The significance of the CCCH-type zinc finger (CTZF) gene in cancer has been increasingly demonstrated during the past few years. According to studies, targeted radical therapy for cancer treatment may be a revolutionary therapeutic approach. Both lncRNAs and CCCH-type zinc finger genes are essential in ccRCC. However, the predictive role of long non-coding RNA (lncRNA) associated with the CCCH-type zinc finger gene in ccRCC needs further elucidation. This study aims to predict patient prognosis and investigate the immunological profile of ccRCC patients using CCCH-type zinc finger-associated lncRNAs (CTZFLs).

**Methods:** From the Cancer Genome Atlas database, RNA-seq and corresponding clinical and prognostic data of ccRCC patients were downloaded. Univariate and multivariate Cox regression analyses were conducted to acquire CTZFLs for constructing prediction models. The risk model was verified using receiver operating characteristic curve analysis. The Kaplan-Meier method was used to analyze the overall survival (OS) of high-risk and low-risk groups. Multivariate Cox and stratified analyses were used to assess the prognostic value of the predictive feature in the entire cohort and different subgroups. In addition, the relationship between risk scores, immunological status, and treatment response was studied.

**Results:** We constructed a signature consisting of eight CTZFLs (LINC02100, AC002451.1, DBH-AS1, AC105105.3, AL357140.2, LINC00460, DLGAP1-AS2, AL162377.1). The results demonstrated that the prognosis of ccRCC patients was independently predicted by CTZFLs signature and that the prognosis of high-risk groups was poorer than that of the lower group. CTZFLs markers had the highest diagnostic adequacy compared to single clinicopathologic factors, and their AUC (area under the receiver operating characteristic curve) was 0.806. The overall survival of high-risk groups was shorter than that of low-risk groups when patients were divided into groups based on several clinicopathologic factors. There were substantial differences in immunological function, immune cell score, and immune checkpoint expression between high- and low-risk groups. Additionally, Four agents, including ABT737, WIKI4, afuresertib, and GNE 317, were more sensitive in the high-risk group.

**Conclusion:** The Eight-CTZFLs prognostic signature may be a helpful prognostic indicator and may help with medication selection for clear cell renal cell carcinoma.

## Introduction

Renal cell carcinoma (RCC) is a cancer with the highest incidence in the urinary system, characterized by high-grade malignancies (Miller et al., 2019). According to morphological classification, RCC can be divided into several subtypes: KIRC, KIRP, and suspicious cell malignancies. KIRC accounts for more than 70% of RCC cases (Fernández-Pello et al., 2017). Despite advances in treatment strategies, the 5-year overall survival (OS) rate (OS) of patients with metastatic clear cell RCC (ccRCC) is only 12% (Siegel et al., 2018). Although significant progress has been made in diagnostic techniques and targeted therapy, the prognosis of most patients remains poor (Fernández-Pello et al., 2017; Gao et al., 2020). The high incidence and recurrence rate of ccRCC emphasizes the urgency of finding novel molecular targets for disease treatment. Combining multiple molecules will significantly improve the accuracy of prognosis prediction.

As a subset of the protein superfamily with the distinctive zinc finger structure, CCCH-type zinc finger proteins are found throughout organisms and play a significant role in nearly every aspect of biological development. The zinc finger protein C2H2 is currently the most researched, while CCCH-type zinc finger protein is rarely studied (Brown, 2005; Hall, 2005). The impacts on plant growth and development have been the main subject of previous investigations on zinc finger genes of the CCCH-type. Recent research has revealed a link between the CCCH-type zinc finger protein and cancer development, incidence, and immunological control. Gastric cancer advancement is controlled by ZC3H15’s targeting of the FBXW7/c-Myc pathway (Hou et al., 2022a), and the progression of colorectal cancer is slowed down by MCPIP3’s suppressive role (Suk et al., 2018a). Through tandem CCCH-type zinc finger RNA, ZFP36 controls AU-rich mRNA’s stability and prevents breast cancer growth (Al-Souhibani et al., 2010). ZC3H13 prevents rectal cancer from spreading and invading by inhibiting the Ras-ERK signaling pathway (Zhu et al., 2019). In a cyclin d-dependent but p53-independent way, ZFP36L1, and ZFP36L2 prevent the proliferation of human colon cancer cells (Suk et al., 2018b). Poor prognosis is linked to high ZC3H15 expression in glioblastoma and melanoma (Li et al., 2021; Hou et al., 2022b). Additionally, Cys-Cys-Cys-His (CCCH)-containing zinc finger proteins have been demonstrated to prevent mRNA stability in immune cells in vitro knockout mice studies (Maeda and Akira, 2017). MCPIP1, 2, 3, and four can control macrophage activation (Liang et al., 2008).

Noncoding RNAs longer than 200 nucleotides are known as long noncoding RNAs (lncRNAs). Thus far, it has been widely proven that lncRNA plays a crucial regulatory role in the development of cancer and many other disease processes (Gibb et al., 2011). Noncoding RNA (ciRS-7) accelerates the growth and metastasis of RCC by triggering the PI3K/AKT signaling pathway (Mao et al., 2021). LINC00973 positively regulates Siglec-15 to take part in the immunological escape response of ccRCC (Liu et al., 2020b, 15). By blocking the miR-27a-3p/FOXO1 axis, ADAMTS9-AS2 can prevent the proliferation of ccRCC cells and decrease their chemoresistance (Song et al., 2019). Recent research has demonstrated that the CCCH-type zinc finger gene ZC3H12D can influence the prognosis of LUAD patients through influencing mRNA, miRNA, lncRNA, immune cells, and immunological components (Chen et al., 2022). In ccRCC, however, the predictive significance of lncRNA linked with the CCCH type of zinc fingers is unknown.

In the current investigation, we developed a predictive signature based on lncRNAs associated with CCCH-type zinc finger genes. The signature performed well in the classification of immunological characteristics and medication selection.

## Materials and methods

### Data collection and processing

We downloaded renal clear cell carcinoma (TCGA-KIRC) RNA-seq data adjusted by FPKM and related clinical and prognostic data from the TCGA website (https://portal.gdc.cancer.gov/); From 613 patients, information on lncRNA expression and survival time was gathered. Data on 111 KIRC patients’ disease-free survival (DFS) were downloaded from the cBioPortal database (https://www.cbioportal.org/). GeneCards (https://www.genecards.org) was used to download 288 genes connected to CCCH-type zinc finger genes. Patients who had been followed up for more than 30 days met the inclusion criterion, and a total of 509 patients were included in the study. In a 1:1 ratio, the patients were split into a training group (n = 256) and a testing group (n = 254).

### Functional enrichment analysis of differentially expressed CCCH-type zinc finger genes

According to previously documented methods (Li et al., 2020), the data was further preprocessed with the limma program with a false discovery rate (FDR) < 0.05 and |log2 fold change (FC)|≥1, and finally, 36 DECZFGs for additional analysis. Three domains were covered by GO analysis: biological processes (BP), cellular elements (CC), and molecular activities (MF). The biological activities of the target genes for CC, MF, and BP were discovered using the GO database (Ashburner et al., 2000). Biological pathway information analysis frequently uses the Kyoto Encyclopedia of Genes and Genomes (KEGG) database (https://www.kegg.jp/), which incorporates genomic, chemical, and system functional information (Kanehisa, 2000). Both GO, and KEGG analyses were performed using the R clusterProfiler package.

### Construction of predictive features of lncRNAs associated with DECZFGs

We calculated the association of zinc finger-related genes with lncRNAs using the “limma” package. Using screening criteria with correlation coefficients |R2 | > 0.3 and *p* < 0.001, CCCH-type zinc finger-associated lncRNAs were discovered. We first performed a univariate Cox regression analysis to obtain lncRNAs connected to the prognosis of ccRCC patients. Then, predictors were chosen, and overfitting was prevented using Least Absolute Shrinkage and Selection Operator (LASSO) regression. The final candidates implicated in the risk signature were then found using multivariate Cox regression analysis. Risk score is calculated as follows: coef (lncRNA1) expr (lncRNA1) + coef (lncRNA2) expr (lncRNA2) +... + coef (lncRNAn) expr (lncRNAn). The coefficient connected to lncRNAs’ survival is known as coef (lncRNAn). lncRNA expression is defined as expr (lncRNAn). CcRCC patients were separated into high-risk and low-risk groups based on the median risk score, and survival and survminer R software packages were used to examine survival differences between the two groups. Principal component analysis (PCA) was used to visualize the grouping ability of risk features using “Limma” and “scatterplot3d” packages.

### Construction of nomograms

We created nomogram survival plots that could predict the 1-year, 2-year, 3-year, and 5-year survival of patients with ccRCC using the risk score in combination with age, gender, grade, stage, M stage, and riskScores. Clinicopathological parameters. We then used calibration curves to determine whether the predicted survival rate was compatible with the actual survival rate.

### Immune infiltrate analysis

Infiltration scores of 16 immune cells and activities of 13 immune-related pathways were calculated using the “GSVA” software package by single-sample gene set enrichment analysis (ssGSEA) (Rooney et al., 2015). The association between risk score and immunological checkpoints was examined by identifying changes in gene expression levels between high and low-risk groups.

### Drug sensitivity analysis of predictive features

We used the Genomics of Drug Sensitivity in Cancer (GDSC) database, a public dataset collecting cancer cell drug sensitivity information and molecular indicators of drug response, to evaluate the role of predictive characteristics in predicting ccRCC treatment response (Iorio et al., 2016). The oncoPredict program was used to download GDSC2 gene expression profiles and associated drug response data (Maeser et al., 2021). The half-maximal inhibitory concentration (IC50) of each medication in patients with ccRCC was predicted using the sensitivity ratings.

### Statistical analysis

R software (version 4.2.1) was used to perform all statistical analyses. The expression levels of DECZFGs in both normal and cancerous tissues were compared using the Wilcoxon test. The Kaplan-Meier technique and the log-rank test analyzed overall survival (OS) in high-risk and low-risk groups. The “survivalROC” software was used to generate ROC curves, and the area under the curve (AUC) was calculated. CCCH-type zinc finger-related lncRNA expression patterns for ccRCC samples were categorized using principal component analysis to show the spatial distribution of high- and low-risk samples (Li et al., 2017; Kim et al., 2018). The “gsva” software was used to perform the ssGSEA analysis.

## Results

### Enrichment analysis of differentially expressed CCCH-type zinc finger-related genes

**FIGURE 1:**
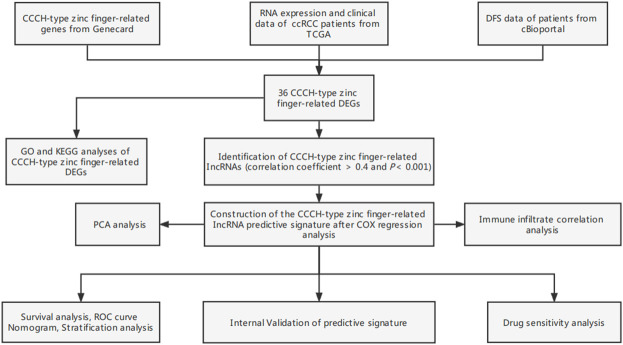
Study flow chart. ccRCC, clear cell renal cell carcinoma; TCGA, Cancer Genome Atlas; DFS, disease-free survival; DEGs, differentially expressed genes; GO, gene ontology; KEGG, Kyoto Encyclopedia of Genes and Genomes; lncRNAs, long-chain non-coding RNA; ROC, receiver operating characteristic; GSEA, gene enrichment analysis; ssGSEA, single-sample gene set enrichment analysis.

**FIGURE 2:**
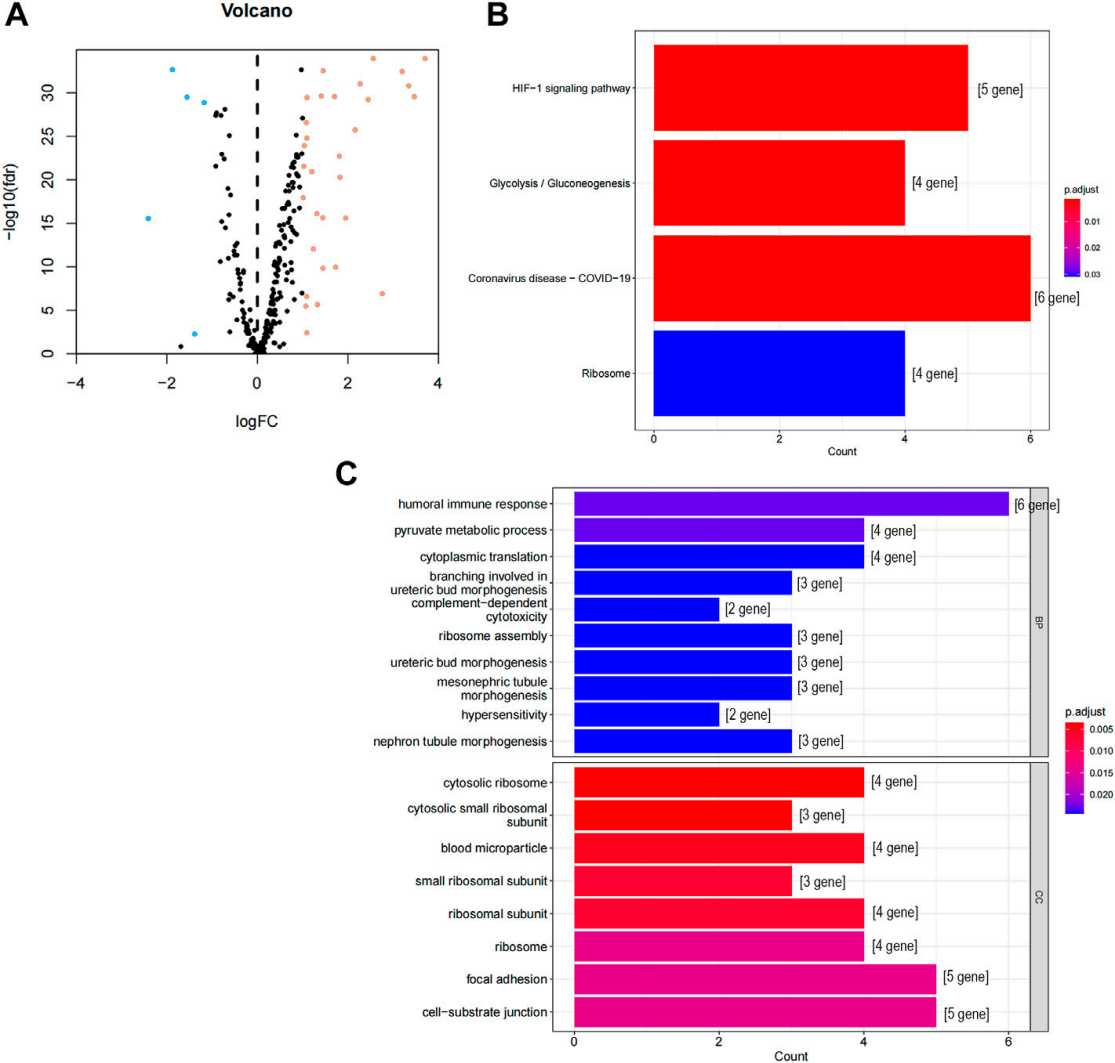
GO and KEGG analysis of CCCH-type zinc finger-related DEGs in cancer and adjacent tissues. **(A)** 262 CCCH-type zinc finger-related genes in ccRCC. Yellow dots indicate up-regulated genes and blue dots indicate down-regulated genes. **(B)** KEGG analysis of CCCH-type zinc finger-related DEGs. **(C)** GO analysis of CCCH-type zinc finger-related DEGs. GO, gene ontology; KEGG, Kyoto Encyclopedia of Genes and Genomes; DEGs, differentially expressed genes; FC, fold changes; fdr: false discovery rate; BP, biological process; CC, cellular composition.

We screened 36 CCCH-type zinc finger-related differentially expressed genes (DEGs), as shown in Figure 1's flowchart, consisting of 31 up-regulated genes and five down-regulated genes (Figure 2A; Supplement file 1). We next carried out a GO and KEGG enrichment analysis of CCCH-type zinc finger-related DGEs. and we found that these DGEs were primarily enriched in the HIF-1 signaling pathway, the glycolysis/gluconeogenesis signaling pathway, the COVID 19 signaling pathway, and the ribosome-related signaling network (Figure 2B). In the category of cellular components, GO analysis revealed that DEGs were mainly enriched in cytoplasmic ribosomes, cytoplasmic small ribosomal subunits, and small ribosomal subunits, as well as humoral immune response, pyruvate metabolism process, cytoplasmic translation, complement-dependent cytotoxicity, and hypersensitivity (Figure 2C).

A predictive signature was developed utilizing differentially expressed CCCH-type zinc finger-related lncRNAs.

**FIGURE 3:**
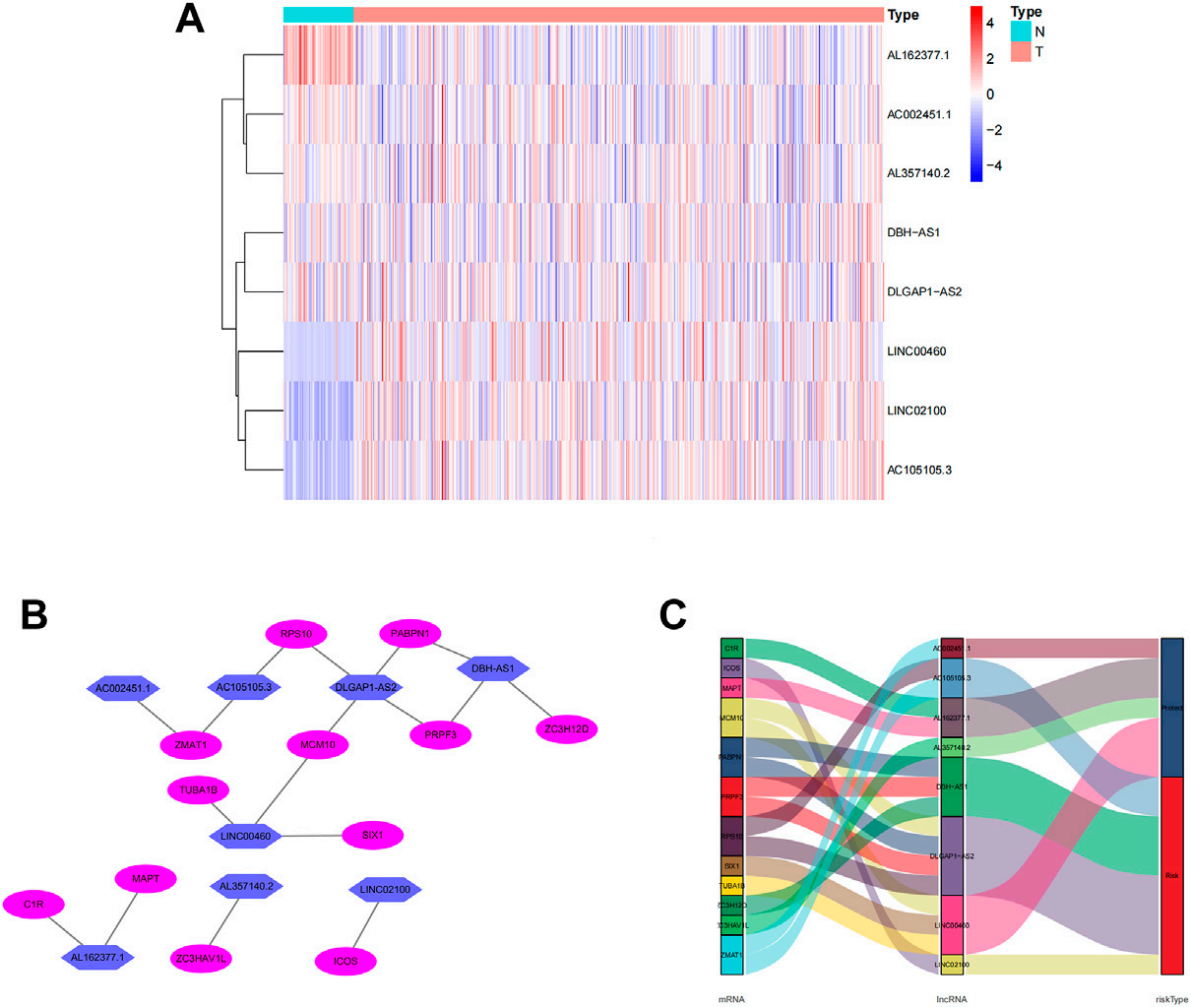
Expression levels of eight CCCH-type zinc finger-associated lncRNA and lncRNA-mRNA networks in predicted signals. **(A)** Expression levels of eight CCCH-type zinc finger-associated lncRNA in ccRCC and normal tissues. **(B)** Co-expression networks of prognostic CCCH-type zinc finger-associated lncRNAs. **(C)** Multinograms of prognostic CCCH-type zinc finger-associated lncrna. LncRNAs, long-chain non-coding RNAs; ccRCC, renal clear cell carcinoma; N, normal; T, tumor.

The screening criteria of correlation coefficients |R2 | > 0.3 and *p* < 0.001 resulted in the identification of 22,79 associated lncRNAs. According to the findings of a univariate Cox regression analysis, 265 lncRNA were linked with the prognosis of ccRCC patients. Eight lncRNAs (LINC02100, AC002451.1, DBH-AS1, AC105105.3, AL357140.2, LINC00460, DLGAP1-AS2, AL162377.1) connected to differentially expressed CCCH-type zinc finger-related were obtained by multivariate regression analysis and used to create predictive characteristics. The eight lncRNAs expression levels in ccRCC patients were observed (Figure 3A). We used the Cytoscape and ggalluvial R software packages to visualize the results further. The co-expression network presented the results for 18 pairs of lncRNA-mRNAs (Figure 3B). AC002451.1 was co-expressed with ZMAT1, AC105105.3 was co-expressed with ZMAT1 and RPS10, DLGAP1-AS2 was co-expressed with MCM10 and PABPN1, DBH-AS1 was co-expressed with PRPF3 and ZC3H12D, LINC00460 was co-expressed with TUBA1B, SIX1 and MCM10, AL162377.1 was co-expressed with C1R and MAPT, AL357140.2 was co-expressed with ZC3HAV1L, and LINC02100 was co-expressed with ICOS. Among these, AC002451.1, AL162377.1, AL357140.2, and LINC00460 were protective factors, and AC105105.3, DBH-AS1, and DLGAP1-AS2 were risk factors (Figure 3B). The risk score was calculated as follows: risk score = (0.181 × LINC02100 expression) + (-0.665 × AC002451.1 expression) + (0.285 × DBH-AS1 expression) + (0.432 × AC105105.3 expression) + (-0.974 × AL357140.2 expression) + (-0.204 × LINC00460 expression) + (0.622 × DLGAP1-AS2 expression) + (-0.852 × AL162377.1 expression).

### The relationship between prognosis and predictive signature in individuals with ccRCC

**FIGURE 4:**
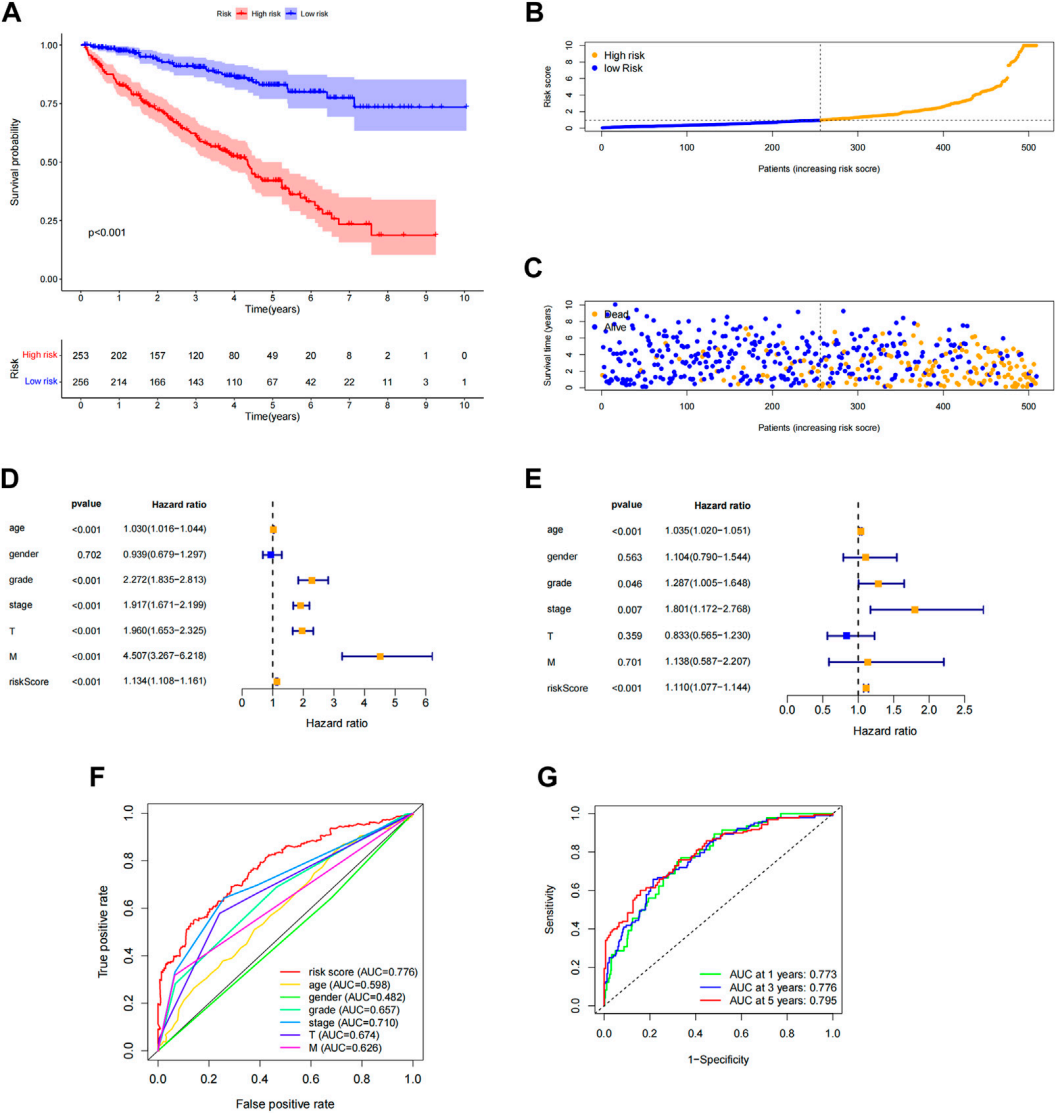
Association of predictive features with prognosis in ccRCC patients. **(A)** Kaplan-Meier analysis of OS rates in the high- and low-risk groups of ccRCC patients. **(B)** Distribution of risk scores in ccRCC patients. **(C)** Number of deaths and surviving patients with different risk scores. Blue blot indicates the number of survivors and yellow blot indicates the number of deaths. **(D)** Forest plot of univariate Cox regression analysis. **(E)** Multivariate Cox regression analysis forest plot. **(F)** ROC curve of risk score and clinicopathological variables. **(G)** ROC curve and AUCs of predictive characteristics of 1-year, 3-year and 5-year survival rates. ccRCC, renal clear cell carcinoma; OS, survival rate; ROC, receiver operating characteristics; AUC, area under the curve; T, tumor.

**FIGURE 5:**
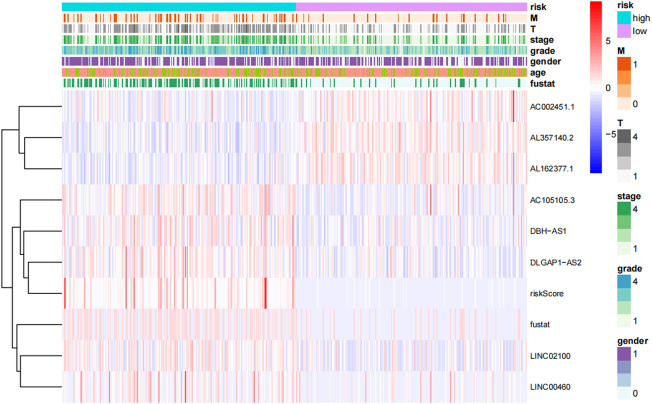
Heat map of the distribution of eight prognosis-related lncrna and clinicopathological variables in the high-risk and low-risk groups. lncRNAs, long-chain non-coding RNAs; M, metastasis; T, tumor.

Each patient’s risk score was determined using the algorithm, and then patients were stratified into high-risk and low-risk categories based on the median score. Kaplan-Meier analysis was used to compare OS between the two groups, and the findings showed that the low-risk group had a significantly longer OS than the high-risk group (Figure 4A, *p* < 0.001). The variance in the risk score was displayed in (Figure 4B), and it was clear that an increase in the risk score was directly correlated to a rise in fatalities (Figure 4C). In order to determine whether the risk characteristics were independent risk factors for the prognosis of ccRCC patients, univariate Cox regression analysis showed that age, grade, stage, T stage, M stage, and risk score were significantly correlated with the OS of ccRCC patients (Figure 4D). Multivariate Cox regression analysis demonstrated that age, grade, stage, and risk score were independent predictors of OS of ccRCC patients (Figure 4E). The predictive value of other clinicopathological indicators was lower than the risk score’s area under the curve (AUC = 0.776). (Figure 4F). The AUC of 1-, 3-, and 5-year survival displayed Strong predictive power. The areas were, correspondingly, 0.773, 0.776, and 0.795. (Figure 4G). We also examined the variations in clinicopathological characteristics between the high-risk and low-risk groups to exclude the influence of these factors. However, wewe were unable to detect any significant differences between the high-risk and low-risk groups (Figure 5).

**FIGURE 6:**
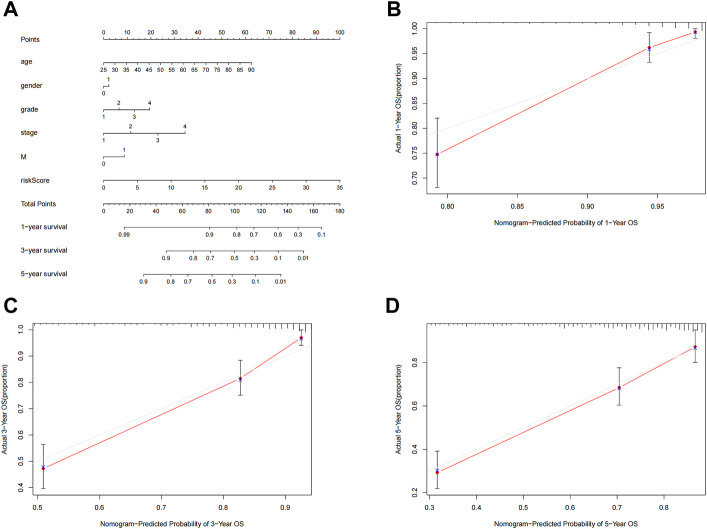
Nomogram construction and validation. **(A)** Nomogram survival combined with clinicopathological factors and risk score predicts 1-year, 3-year, and 5-year survival in ccRCC patients. **(B–D)** Calibration curve tests the consistency between actual OS rate and 1-year, 3-year and 5-year predicted survival. OS, overall survival; ccRCC, renal clear cell carcinoma.

To further predict the prognosis of ccRCC patients, we created nomogram prediction maps incorporating clinicopathological factors and risk scores to forecast the prognosis of ccRCC patients for one, three, and five years (Figure 6A). The actual OS and anticipated survival rates have an excellent correlation after calibration (Figures 6B–D).

### Relationship between the prognosis and other clinicopathologic markers in patients with ccRCC

**FIGURE 7:**
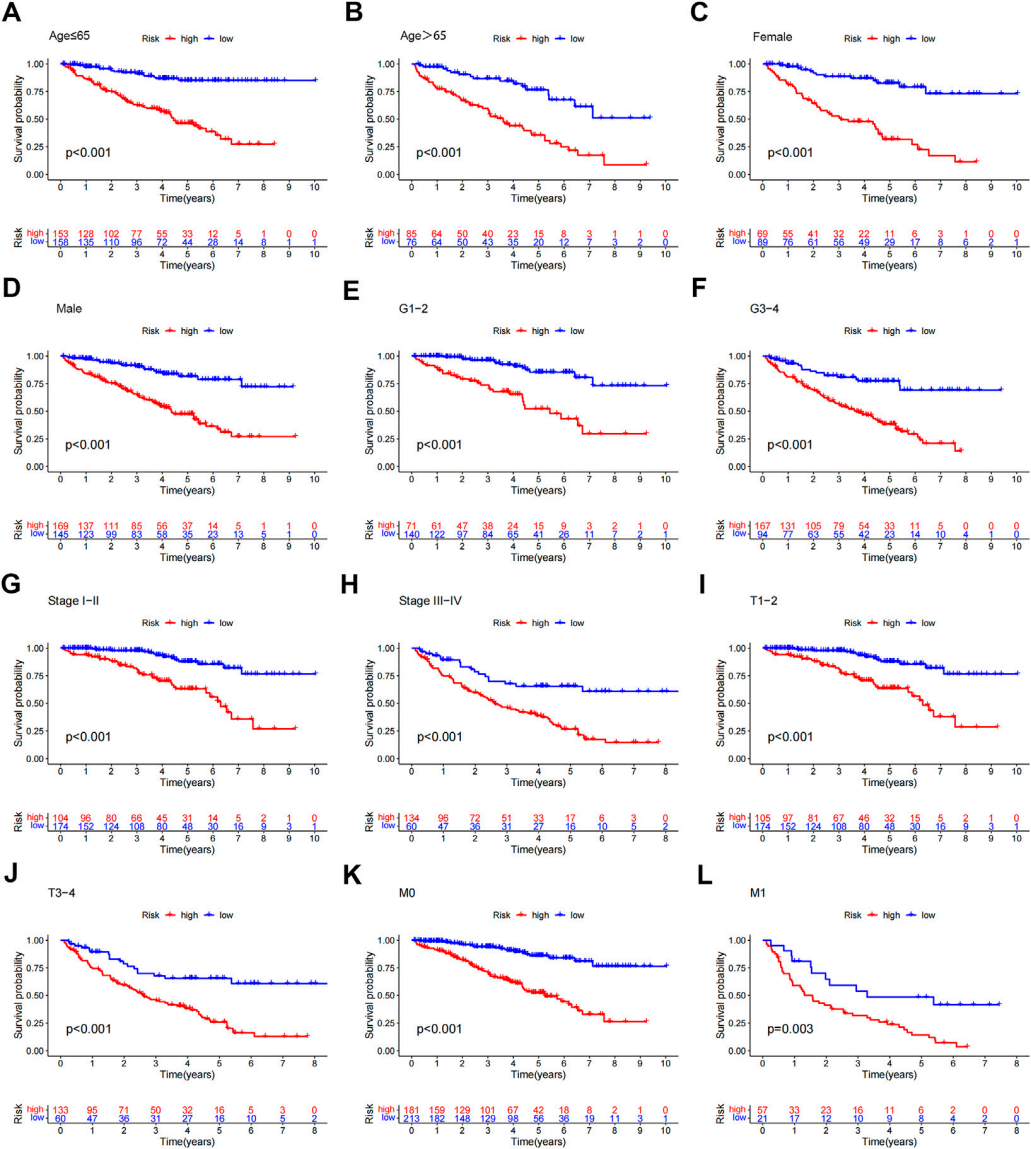
Kaplan-Meier survival curves for patients divided into high- and low-risk groups according to the ranking of different clinicopathological variables. **(A–B)** Age. **(C–D)** Gender. **(E–F)** Grade. **(G–H)** Stage. **(I–J)** T Stage. **(K–L)** M Stage. T, tumor; M, distant metastasis.

To study the relationship between the predictive signature and the prognosis of ccRCC patients sorted according to different clinicopathological variables. CcRCC patients were separated into groups according to age, sex, grade, stage, T stage and M stage, and the prognosis and predictive traits of the various groups were compared. The OS of the high-risk group was considerably lower than that of the low-risk group. These findings suggested that the predictive characteristics might predict the prognosis of ccRCC patients under various clinicopathologic variables (Figure 7).

### Validation of predictive features

**FIGURE 8:**
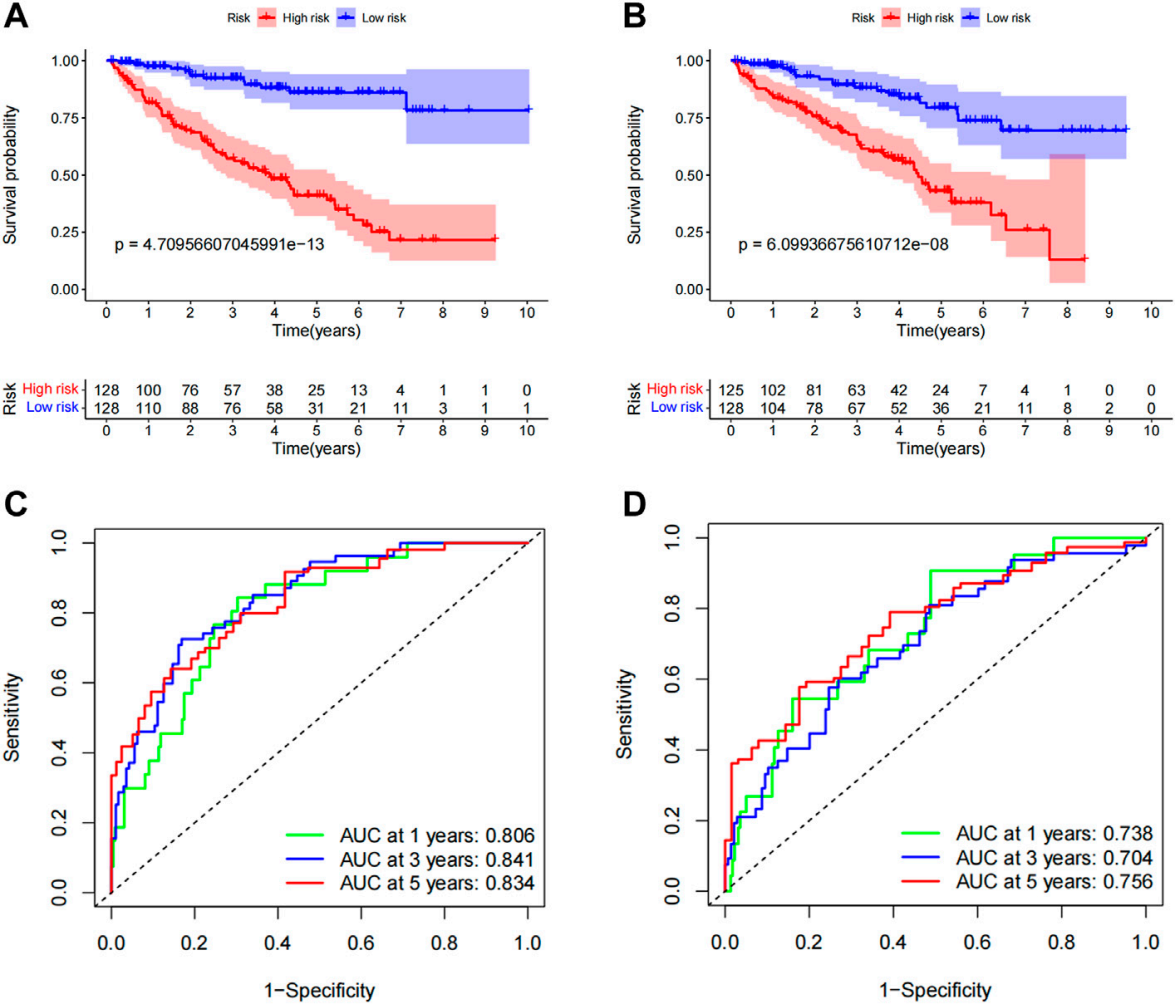
Internal validation of OS prediction signatures based on the TCGA dataset. **(A)** Kaplan-Meier survival curves for the training group. **(B)** Kaplan-Meier survival curves for the testing group. **(C)** ROC curves and AUCs for 1-, 3-, and 5-year survival rates for the training group. **(D)** ROC curves and AUCs for 1-, 3-, and 5-year survival rates for patients in the testing group. ROC, receiver operating characteristic; AUC, area under the curve; OS, overall survival; TCGA, Cancer Genome Atlas.

To evaluate the applicability of the predictive signature, ccRCC patients were randomly separated into two cohorts. The demographic characteristics of the two groups are provided in Table 1, which is consistent with the results seen in the overall dataset. In the training group, the high-risk group was worse than the low-risk group (Figure 8A, *p* = 4.71e 13). The OS rate in the high-risk group in the testing group was lower than that in the low-risk group (Figure 8B, *p* = 6.10e 08). It is possible to see the clinical manifestations of the patients by looking at the ROC curves of the two groups. The 1-year, 3-year, and 5-year survival rates in the training group had AUCs of 0.806, 0.841, and 0.834, respectively (Figure 8C). The 1-year, 3-year, and 5-year survival rates in the testing group were 0.738, 0.704, and 0.756, respectively (Figure 8D). These results suggest that predictive signatures may serve as good indicators of prognosis in ccRCC patients.

**TABLE 1:** The clinicopathologic features characteristics of patients in different cohorts.

Variables	Entire TCGA dataset (n = 509)	Internal validation cohort	
		First cohort (n = 256) second cohort (n = 253)	
Age (%)			
≤65	337 (66.2)	178 (69.5)	159 (62.8)
>65	172 (33.8)	78 (30.5)	94 (37.2)
Gender (%)			
Female	175 (34.4)	80 (31.2)	95 (37.5)
Male	334 (65.6)	176 (68.8)	158 (62.5)
Grade (%)			
G1+2	228 (44.8)	116 (45.3)	112 (44.3)
G3+4	273 (53.6)	136 (53.1)	137 (54.1)
Unknow	8 (1.6)	4 (1.6)	4 (1.6)
Stage (%)			
I + II	307 (60.3)	155 (60.5)	152 (60.1)
III + IV	199 (39.1)	98 (38.3)	101 (39.1)
TX + Unknow	3 (0.6)	3 (1.2)	0 (0.0)
T (%)			
T1 + 2	325 (63.9)	167 (65.2)	158 (62.5)
T3 + 4	184 (36.1)	89 (34.8)	95 (37.5)
M (%)			
M0	402 (79.0)	202 (78.9)	200 (79.1)
M1	79 (15.5)	40 (15.6)	39 (15.4)
MX + Unknow	28 (5.5)	14 (5.5)	14 (5.5)
N (%)			
N0	226 (44.4)	115 (44.9)	111 (43.9)
N1	16 (3.1)	11 (4.3)	5 (1.9)
NX	267 (52.5)	130 (50.8)	137 (54.2)

T, tumor; M, metastasis; N, lymph node.

### Immune cell infiltration and functional analysis

**FIGURE 9:**
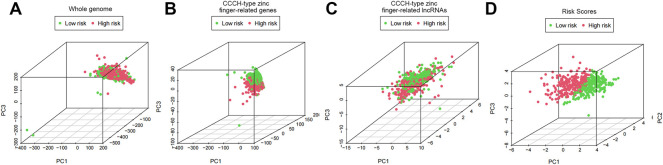
PCA profiles showed patient distribution based on **(A)** Whole genome; **(B)** CCCH-type zinc finger-related genes; **(C)** CCCH-type zinc finger-related lncRNAs; and **(D)** Risk Scores. In the high and low risk groups, red and green dots were more strongly separated.

**FIGURE 10:**
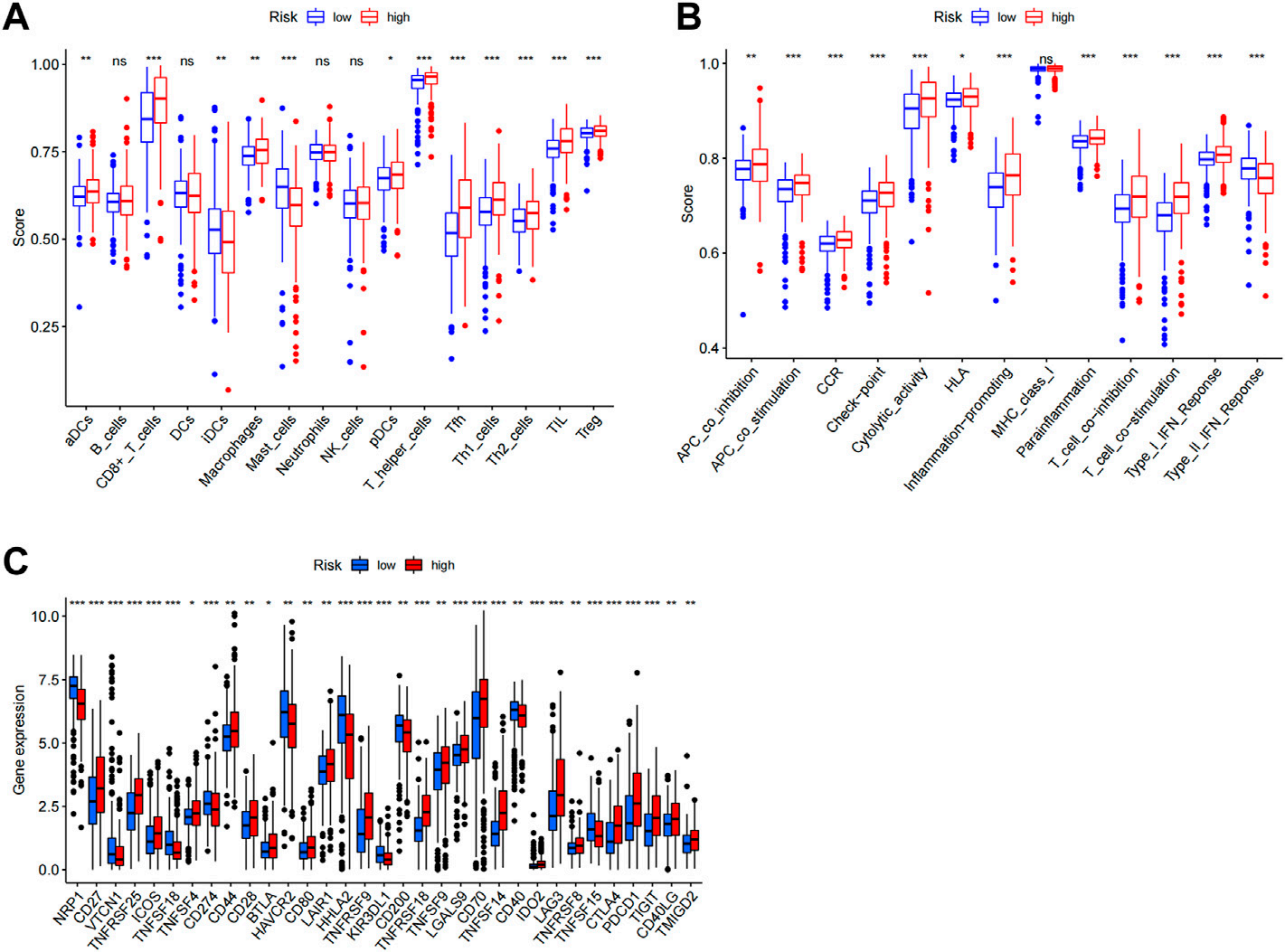
Immune infiltration analysis. ssGSEA score results. A, B Results for ssGSEA scores [immune cells scores **(A)** and immune functions scores **(B**)] between high and low-risk groups in boxplots. **(C)** Expression of immune checkpoints among high and low-risk groups. ns not significant; *p < 0.05; **p < 0.01.

In order to visualize the spatial distribution of high- and low-risk samples, principal component analysis was used to classify the expression patterns of CCCH-type zinc finger-associated lncRNA in ccRCC samples. In order to depict the distribution of patients based on genome-wide, CCCH-type zinc finger-associated gene sets, CCCH-type zinc finger-associated lncRNAs, and risk models, we employed PCA profiles. According to the findings, the risk model was the most beneficial for patients (Figure 9). To investigate how risk scores are related to the immune system, we used ssGSEA to enrich immune cell subsets, and related functions, and we found that patients in the high and low-risk groups had significantly different levels of activated dendritic cells (aDCs), immature dendritic cells (iDCs), CD8 + T cells, plasmacytoid dendritic cells (pDCs), T helper cells, Follicular helper T cell (Tfh), Tumor-infiltrating lymphocytes (TIL), regulatory T cells (Tregs), T helper type 1 (Th1), T help type 2 (Th2) (Figure 10A). Antigen-presenting cells (APCs) co-inhibition, APC co-stimulation, chemokine receptor (CCR), checkpoint, cytolytic activity, human leukocyte antigen (HLA), inflammation-promoting accessory cell, inflammation, and inflammation promoting accessory cell T cell co-inhibition, T cell co-stimulation and immune function scores of type I IFN response is higher in the high-risk group (Figure 10B), which indicated that the immune function of the high-risk group was active. Further exploration revealed that there were also differences in the expression of immune checkpoints between the two groups. We discovered that the high-risk group’s checkpoints all tended to be strongly expressed. These findings strongly suggest that prognostic signature may be closely related to tumor immunity (Figure 10C).

### Relationship between ccRCC therapy and the predictive signature

**FIGURE 11:**
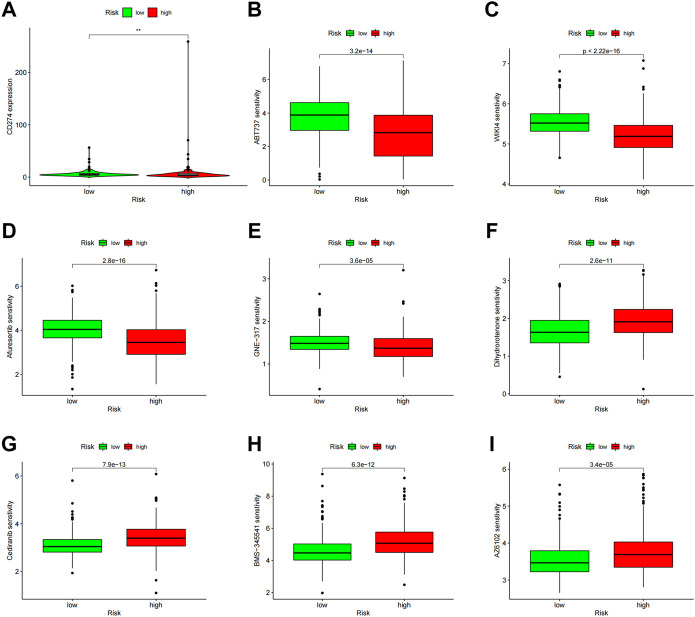
Comparison of treatment drug sensitivity between high- and low-risk groups. **(A)** PD-L1 expression in high and low-risk groups. **(B–E)** Predicted sensitivity of ABT737, WIKI4, Afuresertib, and GNE-317, which were candidate chemotherapeutic agents for high-risk patients. **(F–I)** Predicted sensitivity of Dihydrorotenone, Cediranib, BMS-345541 and AZ6102, which were candidate potent drug options for low-risk patients. PD-L1, programmed cell death ligand 1.

PD-L1 expression was higher in the high-risk group than in the low-risk group, indicating that anti-PD-1/L1 immunotherapy may work for high-risk patients (Figure 11A). Along with immunotherapy, we investigated the relationship between the prediction signature and the effectiveness of conventional treatment for ccRCC. The findings revealed that the high-risk group had higher IC50s for ABT737, WIKI4, Afuresertib, and GNE−317 (Figures 11B–E), whereas the high-risk group had lower IC50s for Dihydrorotenone, Cediranib, BMS−345,541 and AZ6102 (Figures 11F–I). These findings are helpful in examining customized treatment plans for patients in the high- and low-risk groups.

### Construction of CCCH-type zinc finger-associated lncRNAs’ anticipated DFS characteristics

**FIGURE 12:**
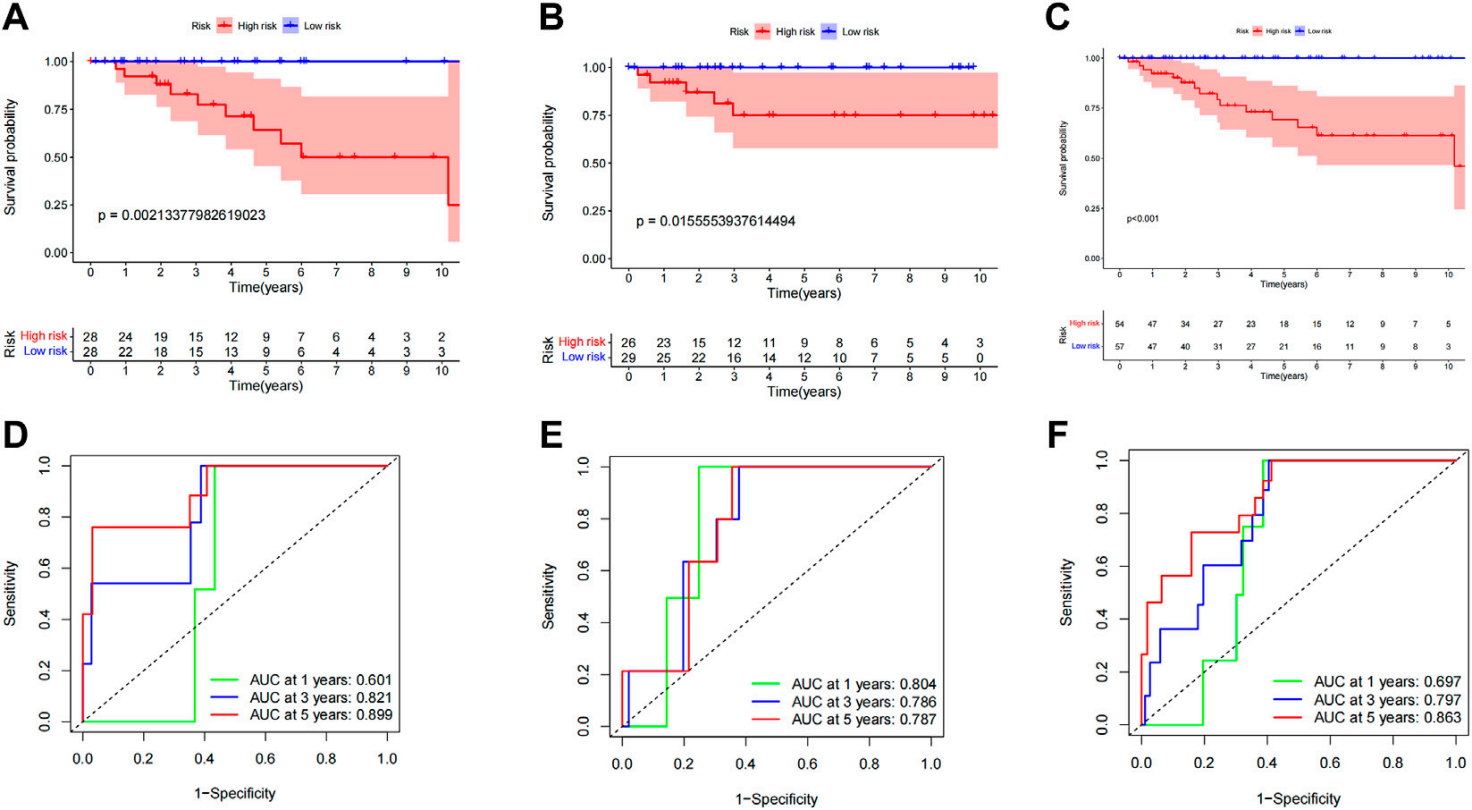
Evaluate the predictive value of CCCH-type zinc finger-associated lncRNA signaling for DFS. (A) Kaplan-Meier survival curves for the entire dataset. **(B)** Kaplan Meier survival curves for the first cohort of patients. **(C)** Kaplan-Meier survival curves for the second cohort. **(D)** ROC curves and AUCs for the 1-, 3-, and 5-year survival rates of the entire dataset. **(E)** ROC curves and AUCs for the 1-, 3-, and 5-year survival rates of the first cohort of patients. **(F)** ROC curves and AUCs for the 1-, 3-, and 5-year survival rates of the second cohort of patients. lncRNAs, long-chain non-coding RNAs; DFS, disease-free survival; ROC: receiver operating characteristic; AUC, area under the curve.

We also constructed a DFS prediction signature lncRNA connected to a CCCH-type zinc finger to account the prognostic significance of disease-free survival (DFS) in ccRCC patients. We obtained DFS information from the cBioPortal database for 111 individuals with ccRCC. We collected DFS data from 111 ccRCC patients from the cBioPortal database. A total of 12 CTZFLs were significantly associated with DFS in ccRCC patients after univariate Cox regression analysis. Two CTZFLs were obtained to construct predictive characteristics by multivariate Cox regression analysis. The risk score formula was as follows: (1.907 × AC244517.7) + (-3.443 × AC011825.2). The dataset patients were divided into high-risk and low-risk groups according to the median risk score. Kaplan-Meier survival curve analysis showed that DFS in the high-risk group was significantly shorter than that in the low-risk group (Figure 12A, *p* < 0.001). The AUC of 1-, 3-, and 5-year survival rates were 0.697, 0.797, and 0.863, respectively (Figure 12D).

To investigate the applicability of predictive characteristics to DFS, 111 patients were randomly divided into the first internal cohort (n = 56) and the second internal cohort (n = 55). Patients were divided into high-risk and low-risk groups according to the median, consistent with the results obtained using the entire dataset analysis. Patients in the high-risk group in the first internal cohort had shorter DFS (Figure 12B, *p* = 2.134e-03) and the second internal cohort (Figure 12C, *p* = 1.556e-02). In the first internal cohort, the AUCs for 1-, 3-, and 5-year survival rates were 0.804, 0.786, and 0.787, respectively (Figure 12E). In the second internal cohort, the AUCs for 1-, 3-, and 5-year survival were 0.697, 0.797, and 0.863, respectively (Figure 12F).

### Association between risk score/CTZFLs and clinical variables

**FIGURE 13:**
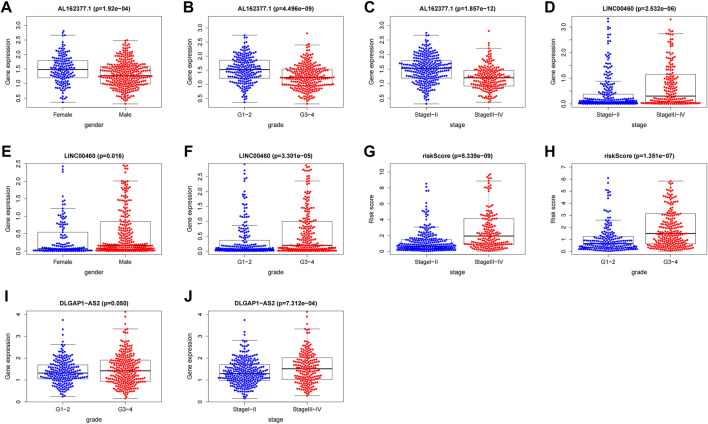
Correlation analysis between CTZFLs and clinical characteristics. **(A–C)** Correlation between AL162377.1 expression level and gender, grade and stage. **(D–F)** Relationship between LINC00460 expression level and gender, stage and grade. **(G–H)** Relationship between risk score and tumor grade and stage. **(I–J)** Relationship between DLGAP1-AS2 expression level and tumor grade and stage. CTZFLs, CCCH-type zinc finger protein-associated lncRNAs.

We investigated the association between clinical variables and risk scores from eight CTZFLs model-based risk scores. The results showed that risk scores were associated with tumor stage and grade; AL162377.1 and LINC00460 were associated with gender, tumor stage, and grade; and DLGAP1-AS2 was associated with grade and stage (Figure 13).

## Discussion

Renal cancer is a heterogeneous disease of unknown etiology. Current evidence suggests that most ccRCC occurs due to several factors, including dysregulation of hypoxia-inducible factor (HIF) signaling, mutations in key histones and chromatin modifying enzymes, and metabolic reprogramming cellular metabolism (Wettersten et al., 2017; Xie et al., 2019). Although diagnostic techniques and targeted therapy have progressed in recent decades, metastasis and invasion can lead to severe poor prognosis in ccRCC and other cancers (Chaffer and Weinberg, 2011). Although sunitinib (RTK) is the first-line treatment for advanced ccRCC, most patients succumb to the disease due to drug resistance (Bergers and Hanahan, 2008; Huang et al., 2010). As bioinformatics technology develops, more and more biomarkers are found that could be used as diagnostic and therapeutic targets for ccRCC. Several biomarkers can increase the precision of prediction outcomes (Tamayo et al., 2011; Ahmed and Abedalthagafi, 2016), but the heterogeneity of ccRCC disease leads to the inability to predict the results by a single molecular marker. At present, the development of multivariate models to indicate the prognosis of cancer has become a research hotspot.

In this study, we obtained 36 DGEs with CTZF for the first time. KEGG analysis showed that DEGs were mainly enriched in the HIF-1 signaling pathway, glycolysis/gluconeogenesis signaling pathway, COVID-19 signaling pathway, and ribosome-related signaling pathway. It has been demonstrated that HIF-1 can hinder miR-32’s upregulation of HECTD2, hence accelerating the progression of renal carcinoma (Lv et al., 2021). A poor prognosis is typically predicted by elevated HIF1 in ccRCC (Gudas et al., 2014). These results suggest that CZF may regulate the progression of ccRCC through the HIF-1 signaling pathway.

By using univariate Cox and LASSO regression analysis, CTZFLs related with prognosis were identified (LINC02100, AC002451.1, DBH-AS1, AC105105.3, AL357140.2, LINC00460, DLGAP1-AS2, AL162377.1). We created a novel prognostic prediction model based on these eight key genes to investigate whether these specific CTZFLs might be employed as prognostic factors. This model is the first CTZFL-related predictive risk model that we are aware of. Univariate and multivariate Cox regression analysis results demonstrated that the risk model is a reliable prognostic indicator for ccRCC. Its biological importance in determining the prognosis of ccRCC was further supported by survival analysis and ROC analysis. Nomogram analysis also revealed a similar circumstance. The total survival of patients with ccRCC could be virtually predicted by risk markers, and they performed examinations considerably more effectively than other clinicopathological factors. These investigations offered fresh ideas for future research and revealed for the first time the prognostic significance of the CTZFs gene-dependent risk model for patients with ccRCC.

Clinical outcomes in renal cell carcinoma (RCC) correlate highly with immune infiltration. The quantity and proportioning of invading immune cells are currently thought to be crucial to cancer development and the efficacy of immunotherapy, and they are directly correlated with patient prognosis. In the tumor microenvironment, tumor-infiltrating immune cells (TIICs) create a tiny ecosystem and exhibit potential prognostic significance (Grivennikov et al., 2010). Tumor growth can be targeted and suppressed by cytotoxic CD8^+^ T cells and CD4^+^ helper T cells (Vesely et al., 2011). However, alterations in the composition of the tumor microenvironment, such as regulatory T cells (Tregs) that can emit immunosuppressive cytokines that impair T cell function, decrease the response of associated T cells, resulting in the loss of immunogenicity of the tumor (Wherry and Kurachi, 2015; Speiser et al., 2016). High-risk patients were found to have strong associations with immune-related pathways, according to the GSEA. In the follow-up ssGSEA, it was discovered that the high-risk group had higher scores for CD8^+^ T cells, macrophages, pDCs, T-helper cells, and Tregs. High CD8 + T cell infiltration has been linked to poor outcomes in BC patients, according to studies (Hou et al., 2020). A poor prognosis is linked to increased tumor-associated macrophage infiltration in advanced thyroid carcinoma (Ryder et al., 2008). High Treg infiltration in hepatocellular carcinoma patients is an adverse prognostic sign (Tu et al., 2016). In RCC, Tregs have been demonstrated to dramatically inhibit the growth of effector T cells (Santagata et al., 2017). According to a study, ccRCC results were negatively impacted by T cell follicular helper cells, T cell regulation, and B cell memory (Yu et al., 2020). The traits of the high-risk group that we have identified are consistent with the research mentioned above and indicate a dismal prognosis for members of the high-risk group. In conclusion, patients with a high-risk score have a poor prognosis that may be attributed to immune system infiltration by macrophages, regulatory T cells (Tregs), and T cell follicular helper cells. Additionally, The high-risk group also showed higher HLA and type I IFN response scores, lower antitumor immunity, and greater tumor immune cell infiltration. Therefore, the poor prognosis may be brought on by the high-risk group’s lower antitumor immunity. Immune checkpoints are significantly expressed differently in high-risk and low-risk populations, suggesting that different populations will respond differently to immunotherapy, and checkpoint inhibitor-based immunotherapy increases survival for many patients with advanced cancers, including renal cancer (Hellmann et al., 2018). Renal cancer patients’ survival following treatment with immune checkpoint inhibitors, such as nivolumab, has increased dramatically due to drug trials (Barata and Rini, 2017), which have also improved the therapeutic outlook for renal cancer.

So far, a growing number of studies have demonstrated the significance of CCCH-type zinc finger protein in the progression of cancerous tumors (Al-Souhibani et al., 2010; Suk et al., 2018a, 2018b; Zhu et al., 2019; Li et al., 2021; Hou et al., 2022a, 2022b). As a result, there has been a rise in interest in its potential application in the prognosis prediction of renal cancer. lncRNAs have been discovered to influence immune cell infiltration and the tumor immune response to influence tumor formation. A new target for sunitinib resistance has been identified: SNHG12, which has been shown in recent trials to increase sunitinib resistance and progression (Liu et al., 2020a). By overexpressing ASS1, LncRNA 00312 can promote apoptosis in RCC cells (Zeng et al., 2020), offering a possible target for RCC treatment. Therefore, based on the predictive characteristics of CTZFLs and targeting lncRNA and Drug sensitivity analysis combination may create a new regimen for the prognosis prediction and treatment of ccRCC.

However, several issues still need to be resolved. First, we only used data from the TCGA database for internal validation, and we still need data from other databases for external validation to test the applicability of the predictive signature. Secondly, we need to build cell and animal models to verify these results using PCR, immunohistochemistry, and western blotting for CTZFLs implicated in model construction.

## Conclusion

In conclusion, CCCH-type zinc finger gene-related lncRNA features can independently predict the prognosis of ccRCC patients and offer a viable strategy for anti-tumor immunotherapy and the choice of chemotherapeutic medicines.

## Data Availability

The datasets presented in this study can be found in online repositories. The names of the repository/repositories and accession number(s) can be found in the article/Supplementary Material.
