# Supplementary material for: A new CCCH-type zinc finger-related lncRNA signature predicts the prognosis of clear cell renal cell carcinoma patients
Source: Front Genet. 2022 Sep 30;13:1034567. doi: 10.3389/fgene.2022.1034567 (PMC9562972; doi:10.3389/fgene.2022.1034567)
Supplement: Supplementary file 2 [file Table1.DOC]

Table 1 | The clinicopathologic features characteristics of patients in different cohorts.

| Variables | Entire  TCGA dataset (n = 509) | Internal Validation cohort  First cohort (n = 256) Second cohort (n = 253) | |
| --- | --- | --- | --- |
| Age (%)  ≤65  ＞65 | 337 (66.2)  172 (33.8) | 178 (69.5)  78 (30.5) | 159 (62.8)  94 (37.2) |
| Gender (%)  Female  Male | 175 (34.4)  334 (65.6) | 80 (31.2)  176 (68.8) | 95 (37.5)  158 (62.5) |
| Grade (%)  G1+2  G3+4  Unknow | 228 (44.8)  273 (53.6)  8 (1.6) | 116 (45.3)  136 (53.1)  4 (1.6) | 112 (44.3)  137 (54.1)  4 (1.6) |
| Stage (%)  I + II  III + IV  TX + Unknow | 307 (60.3)  199 (39.1)  3 (0.6) | 155 (60.5)  98 (38.3)  3 (1.2) | 152 (60.1)  101 (39.1)  0 (0.0) |
| T ( % )  T1 + 2  T3 + 4 | 325 (63.9)  184 (36.1) | 167 (65.2)  89 (34.8) | 158 (62.5)  95 (37.5) |
| M ( % )  M0  M1  MX + Unknow | 402 (79.0)  79 (15.5)  28 (5.5) | 202 (78.9)  40 (15.6)  14 (5.5) | 200 (79.1)  39 (15.4)  14 (5.5) |
| N ( % )  N0  N1  NX | 226 (44.4)  16 (3.1)  267 (52.5) | 115 (44.9)  11 (4.3)  130 (50.8) | 111 (43.9)  5 (1.9)  137 (54.2) |

T, tumor; M, metastasis; N, lymph node.
